# Olives and Bone: A Green Osteoporosis Prevention Option

**DOI:** 10.3390/ijerph13080755

**Published:** 2016-07-26

**Authors:** Kok-Yong Chin, Soelaiman Ima-Nirwana

**Affiliations:** Department of Pharmacology, Universiti Kebangsaan Malaysia Medical Centre, 56000 Cheras, Malaysia; imasoel@ppukm.ukm.edu.my

**Keywords:** menopause, olives, oleuropein, polyphenol, skeleton, tyrosol

## Abstract

Skeletal degeneration due to aging, also known as osteoporosis, is a major health problem worldwide. Certain dietary components confer protection to our skeletal system against osteoporosis. Consumption of olives, olive oil and olive polyphenols has been shown to improve bone health. This review aims to summarize the current evidence from cellular, animal and human studies on the skeletal protective effects of olives, olive oil and olive polyphenols. Animal studies showed that supplementation of olives, olive oil or olive polyphenols could improve skeletal health assessed via bone mineral density, bone biomechanical strength and bone turnover markers in ovariectomized rats, especially those with inflammation. The beneficial effects of olive oil and olive polyphenols could be attributed to their ability to reduce oxidative stress and inflammation. However, variations in the bone protective, antioxidant and anti-inflammatory effects between studies were noted. Cellular studies demonstrated that olive polyphenols enhanced proliferation of pre-osteoblasts, differentiation of osteoblasts and decreased the formation of osteoclast-like cells. However, the exact molecular pathways for its bone health promoting effects are yet to be clearly elucidated. Human studies revealed that daily consumption of olive oil could prevent the decline in bone mineral density and improve bone turnover markers. As a conclusion, olives, olive oil and its polyphenols are potential dietary interventions to prevent osteoporosis among the elderly.

## 1. Introduction

Skeletal mass and microarchitecture degenerate with aging, thus predisposing the elderly to skeletal fragility and fractures. This condition, aptly named ‘osteoporosis’ or bone porosity in Greek, affects both elderly men and women worldwide [1]. The global estimate of osteoporotic hip fracture incidence in 2000 was nine million, and resulted in more disability adjusted life years lost compared to common cancers excluding lung cancers [2]. The prevalence of osteoporosis is predicted to rise exponentially with the accelerated expansion of elderly population, especially in developing countries [3]. The tremendous economic and healthcare burdens caused by osteoporotic fractures deserve much attention from the medical and scientific community. 

Bone homeostasis is regulated by a remodeling process governed by three types of bone cells, i.e., osteoblasts for bone formation, osteoclasts for bone resorption and osteocytes as the mediators of bone remodeling. Osteoporosis is a dysregulation of bone remodeling, whereby the rate of bone resorption is greater than that of bone formation, resulting in a net bone loss [4]. Sex hormone deficiency is the major cause of osteoporosis, but the roles of inflammation and oxidative stress in the pathogenesis of osteoporosis are gaining recognition [5,6,7]. Inflammation cytokines, such as interleukin-1 beta, interleukin-6 and tumour necrosis-alpha, favour the formation of osteoclast, thus promote bone resorption [8]. Consequently, patients with inflammatory diseases are prone to suffer from low bone mass and fractures [9,10]. Free radicals serve as signalling molecules in osteoclasts enhancing their differentiation [11]. Oxidative stress also damages osteoblasts and suppresses their differentiation [12]. Studies have shown that bone mineral density is correlated with oxidative status in humans [13,14]. 

**Figure 1:**
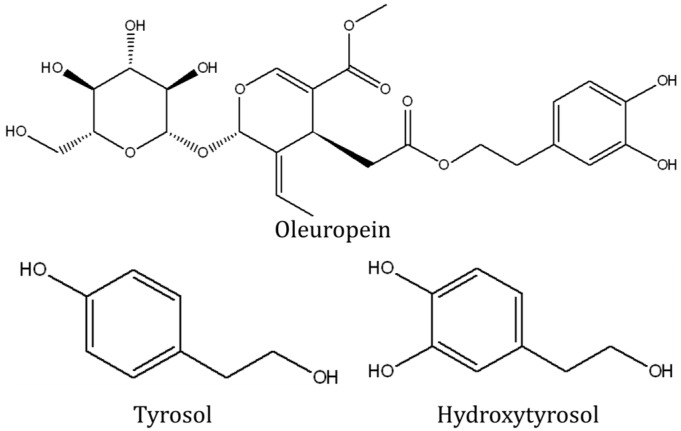
Chemical structure of oleuropein, tyrosol and hydroxytyrosol.

Compounds in our diet possessing antioxidative and anti-inflammatory properties, such as tocotrienol [15,16], curcumin [17], and omega-3 fatty acids [18], demonstrate promising bone protective effects. Adherence to Mediterranean diet was associated with decreased fracture incidence (hazard ratio: 0.93, CI: 0.89–0.98) in the European Prospective Investigation into Cancer and Nutrition Study involving 188,795 subjects (mean age 48.6 years (standard deviation (SD): 10.8 years)) followed for nine years [19]. Mediterranean diet was also associated with increased calcium absorption and retention, and a decrease in urinary calcium excretion in male adolescents (*n* = 20; mean age: 12.9 years (SD: 1.14 years)) [20]. Olives and olive oil are important components in the Mediterranean diet. A Mediterranean diet enriched with olive oil has been associated with increased levels of bone formation markers than non-enriched diet in elderly men (*n* = 125; age 55–80 years) [21]. Results of these human studies have been validated in experimental studies, whereby supplementation of extra virgin or refined olive oil (19% of the diet) in growing pigs for eight weeks increased their bone mineral density gain per day significantly [22]. Rezq et al. found that substituting dietary lipids with olive oil for six weeks increased femoral length, volume, bone mineral density and mineral content in mice [23]. Mice fed with olive oil also had higher apparent calcium absorption and calcium balance, but a lower serum calcium, phosphate and magnesium level compared to groups fed with other lipids [23]. This could be attributed to the high phenolic content of olives. These phenolic compounds, which include tyrosol, hydroxytyrosol and oleuropein, exert prominent antioxidant and anti-inflammatory effects; thus are potential candidate agents for osteoporosis prevention (Figure 1) [24,25]. 

The objective of this review is to summarize the current evidence from cellular, animal and human studies on the effects of olives, olive oil and olive polyphenols (from olive oil and olive mill waste water) on bone health. We have included studies using animal model of osteoporosis, cellular model of osteoblasts and osteoclasts, and human trials to assess the bone protective effects of olives. In these studies, bone health was determined by bone mineral density assessed using dual-X-ray absorptiometry, bone turnover by circulating bone formation and resorption markers, skeletal microarchitecture by histology, histomorphometry or X-ray computed tomography, and bone biomechanical strength. To assess the mechanism of action of olives on bone, oxidative stress and inflammatory markers were used. We aim to provide a comprehensive view on the current evidence of olives, olive oil and its polyphenols as dietary options against osteoporosis. We conclude the review with suggestions of possible future research directions on this topic. 

## 2. Bone Protecting Effects of Olives

Lucques olives produced in southern France are rich in phenolic compounds. Black Lucques olives contain relatively higher levels of hydroxytyrosol (146 mg/100 g fresh weight) and tyrosol (143 mg/100 g fresh weight) compared to green olives (16 mg/100 mg tyrosol and negligible amount of hydroxytyrosol). Puel et al. compared the osteoporosis-preventing effects of black Lucques and green olives in 6-month old ovariectomized rats. Fifty g/kg fat in the diet deficient in vitamin E, D and calcium was substituted with fat from olives (equivalent to 6 g of black Lucques olive or 10 g green Lucques olive per day) and given to the rats for 84 days. Inflammation was induced in these rats using magnesium silicate (3.2 g per animal) at day 63. Diet supplemented with black Lucques olives significantly prevented the decrease of femoral diaphyseal bone mineral density in the inflamed ovariectomized group but this was not seen with green olives. Changes in bone biomechanical strength, markers of bone turnover (formation: osteocalcin; resorption: urinary deoxypyridinoline), inflammation (α-1-acid glycoprotein and fibrinogen) and oxidative stress (15-isoprostane F2t and ferric reducing ability of plasma) were not significant in both groups. However, plasma level of α-tocopherol, an antioxidant, was elevated in both supplemented groups. Calciuria, indicating calcium loss, was found in rats supplemented with green olives. Overall, the osteoporosis preventing effects of the black Lucques olives was superior compared to the green olives. This could be attributed to a higher polyphenol level in the black Lucques compared to the green olives [26]. 

## 3. Bone Protecting Effects of Olive Oil

Diet supplemented with 50 g/kg extra virgin olive oil for 80 days prevented the decrease of femoral total, diaphyseal and metaphyseal bone mineral density in 6-month old ovariectomized rats with induced inflammation, but not in those without inflammation. Although both ovariectomy and inflammation did not cause degeneration in bone strength, extra virgin olive oil increased the femoral failure load in ovariectomized rats with inflammation. However, plasma osteocalcin (a bone formation marker) and urinary deoxypyridinoline (a bone resorption marker) were not affected by the treatment. Supplementation with extra virgin olive oil also decreased the plasma α-1-acid glycoprotein, an inflammation marker, in ovariectomized rats with inflammation. However, oxidative status of the osteoporotic rats, indicated by plasma malondialdehyde and ferric reducing ability, was not altered by the treatment. The effects of 50 g/kg extra virgin olive oil on bone are similar to 0.15 g/kg oleuropein [27].

Saleh and Saleh supplemented 12–14 months old ovariectomized rats with 1 mL/kg body weight extra virgin olive oil for 12 weeks (4 weeks before and 8 weeks after ovariectomy). The treatment significantly prevented the reduction in cortical and trabecular bone thickness assessed using a routine histology technique and activity of alkaline phosphatase. It also prevented the mobilization of calcium reserve in the bone, indicated by a lower plasma calcium level in the treated group compared to ovariectomized control. This might be contributed by the reduction of oxidative stress, indicated by a decline in malondialdehyde and nitrate levels, in treated rats [28]. Proper bone histomorphometry technique was not applied to assess the conventional bone structural, cellular and dynamic parameters in this study.

Liu et al. compared the effectiveness of olive oil supplementation (1 mL/100 g diet) and diethylstilbestrol (25 ug/kg diet), a synthetic oestrogen mimicking oestrogen replacement therapy in humans, for 12 weeks in 6 month old ovariectomized rats. Both treatments increased the bone mineral density of lumbar spine and left femur of the ovariectomized rats. This might be contributed by decreased oxidative stress in the supplemented groups, marked by reduced malondialdehyde and nitrate levels. However, hypophosphatemia (probably due to ovariectomy-induced hyperparathyroidism), increased alkaline phosphate activity (indicative of elevated bone remodeling) and interleukin-6 levels were not prevented by both treatments. This study showed that olive oil was as effective as oestrogen replacement in preventing post-menopause bone loss [29].

## 4. Bone-Protecting Effects of Olive Polyphenols

Olive plants produce a variety of polyphenols, particularly oleuropein, tyrosol and hydroxytyrosol [30,31]. They can be found in various parts of the plants, i.e., in the leaves, the fruits and in the waste water of olive mill as by-products of olive oil production [30,31]. Single olive polyphenol compounds or a mixture of them might protect against bone loss. Oleuropein given in four doses (2.5–15 mg/kg) for 100 days prevented the decline of total, diaphyseal and metaphyseal bone mineral density of the femur in 6-month old inflamed ovariectomized rats. Oleuropein at 15 mg/kg also halted high bone remodeling, indicated by a significant decrease of serum osteocalcin and a marginal decrease of urinary deoxypyridinoline level in inflamed ovariectomized rats. However, oleuropein did not improve biomechanical strength of the bone and ferric reducing ability of the plasma [32]. An earlier study showed that bone strength was only altered by oleuropein at 0.15 g/kg for 80 days [27], implying that the dose used in this study might not be sufficient.

In the study by Hagiwara et al. ovariectomized mice were given hydroxytyrosol, tyrosol or oleuropein at 10 mg/kg orally for 28 days. Hydroxytyrosol and oleuropein treatments increased the femoral trabecular bone mineral density but had no effects on the cortical compartment. The efficacy of hydroxytyrosol was greater compared to oleuropein and this was attributed to a better absorption of hydroxytyrosol in vivo [33].

Olive mill waste water is a by-product of olive oil production containing high levels of polyphenols. In a subsequent study by Puel et al. 6-month old inflamed ovariectomized rats were fed with diet supplemented with 0.017% tyrosol, or 0.017% hydroxytyrosol, or 0.17% olive mill wastewater, or 0.08% or 0.0425% olive mill wastewater extract for 84 days. Supplementation of tyrosol, hydroxytyrosol and two concentrations of olive mill waste water extract prevented the reduction in femoral total, metaphyseal and diaphyseal bone mineral density. Olive mill waste water extract at 0.08% diet was found to increase osteocalcin level in the inflamed ovariectomized rats, but all treatment did not influence level of urinary deoxypyridinoline. Hydroxytyrosol and the two concentrations of olive mill waste water extract also lowered the level of isoprostane, an index of oxidative stress, in inflamed ovariectomized rats. The lower concentration of olive mill waste water also prevented the increase in granulocytes, indicative of inflammation, in the blood of inflamed ovariectomized rats. However, biomechanical strength of the bone, ferric reducing ability of the plasma and plasma fibrinogen levels were not affected by all interventions [31].

Keiler et al. showed that supplementation with the phenolic-rich extract of extra virgin olive oil at 800 mg/kg diet for 12 weeks exerted estrogenic effects in 12-month old ovariectomized rats. Expression of oestrogen responsive genes (estrogen receptor 1 (ESR1), marker of proliferation Ki-67 (Mki67) and complement component 3 (C3)) in the uterus was significantly altered by the treatment, so that their levels were on par with the sham group. Uterine weight was slightly increased in the treated group. However, the phenolic rich fraction did not prevent degeneration of bone structural indices induced by ovariectomy [34]. This could be due to the shorter length of treatment of this study compared to the previous ones. The effects of olives, olive oil and its polyphenols on bone in animal models of osteoporosis have been summarized in Table 1.

## 5. Studies of Olive Polyphenols on Bone Cells Using in Vitro Models

In a cell culture study, Hagiwara et al. demonstrated that hydroxytyrosol, tyrosol and oleuropein (each at concentration between 1 and 100 µM) exerted distinct effects on different types of bone cells. In terms of bone formation, all three polyphenols failed to increase the proliferation and the production of collagen and the activity of alkaline phosphatase (a bone formation marker) in MC3T3-E1 cells, a model of the osteoblast. Oleuropein at 100 µM significantly increased the deposition of calcium in an osteoblasts culture. Hydroxytyrosol also exhibited similar effects but the trend of increased calcium deposition was not statistically significant. In addition, formation of hydrogen peroxide in MC3T3-E1 cells was suppressed by 50 µM of hydroxytyrosol [33].

In terms of bone resorption, oleuropein at 10 µM significantly decreased the formation of tartrate-resistant acid phosphatase positive cells (osteoclast-like cells) from a spleen culture. At 50 and 100 µM, oleuropein completely suppressed the formation of these osteoclast-like cells in vitro. Hydroxytyrosol (50 and 100 µM) and tyrosol (100 µM) also reduced the formation of tartrate-resistant acid phosphatase positive cells in culture [33]. 

A study by Gracia-Martinez et al. examined the effects of phenolic content of various Sicilian virgin olive oils on a cellular model of osteoblasts. The phenolic extract of most olive oils could increase the proliferation of human MG-63 osteosarcoma cells in a non-concentration-dependent manner. However, the efficacy of the extract was independent of the phenolic content of the olive oils, thus implying that other substances were involved in the proliferation of osteoblasts [35]. In their follow-up study, human MG-63 osteosarcoma cells were treated with individual components or total phenolic extract of virgin olive oil of different variety. It was found that hydroxytyrosol, caffeic acid, *p*-coumaric acid, ferulic acid, luteolin and apigenin increased the proliferation of osteoblasts, but the total phenolic content of virgin olive oil from several olive species was better than individual phenolic acid in this regards. This observation suggested there were synergistic effects among phenolic acids present in the olive oil in promoting osteoblastic proliferation. Besides, the proliferation-enhancing effects of phenolic extracts from young fruits was better than ripen fruits due to a higher phenolic content in the unripe fruits [36]. The proliferative effect of olive polyphenol was not shown in the study of Hagiwara et al. [33] probably because of the use of a different osteoblast model. 

In a recent study, oleuropein at the concentration of 1 µM and 100 µM was found to enhance osteoblastogenesis and suppress adipogenesis in human bone marrow culture. The gene expression of osteoblast formation and differentiation markers, i.e., runt-related transcription factor II, osterix, alkaline phosphatase and collagen type I and osteocalcin were up-regulated in a time-dependent manner in a culture supplemented with oleuropein. This was accompanied by an increase in the ratio of osteoprotegerin over receptor activator of nuclear factor kappa-B (NF-κβ) ligand stimulated by oleuropein. Ultimately, the gene modulation effects of oleuropein were translated to an increase in mineral deposition by osteoblasts in the culture. In contrast, expression of genes related to adipogenesis such as peroxisome proliferator-activated receptor gamma 2 (PPARγ2), the lipoprotein lipase (LPL), and fatty acid-binding protein 4 (FABP4) were suppressed by oleuropein. Size of lipid droplets and oil red stain area in the culture were significantly reduced in the bone marrow cultured with oleuropein [37].

## 6. Human Studies on the Bone Protecting Effects of Olive Oil

Three trials were performed to examine the effects of olive oil on bone health in humans. In the “Prevencion con Dieta Mediterranea” (PREDIMED) study, 127 community-dwelling men aged 55–80 years with high cardiovascular risk were divided into three groups taking Mediterranean diet with virgin olive oil (at least 50 mL/day), Mediterranean diet with mixed nuts (at least 30 g/day) or a low-fat diet, and were followed-up for two years. Fernandez-Real et al. observed that circulating total osteocalcin and procollagen type I N-propeptide levels, both markers of bone formation, were increased in men taking Mediterranean diet with virgin olive oil but not in those taking Mediterranean with mixed nuts, while collagen type 1 cross-linked C-telopeptide level, a marker of bone resorption, decreased in all groups. Olive consumption was positively associated with total osteocalcin at baseline (*r* = 0.23; *p* = 0.02) and follow-up (*r* = 0.21; *p* = 0.04), before and after adjusting for the use of statins [21]. This was a cardiovascular study; thus bone health was not the primary end-point. Despite the positive results in bone remodeling markers, it was not known whether olive or olive oil consumption translated to an improved bone mineral density or a reduction in fracture risk. 

In the study of Liu et al., subjects aged 30–50 years who had undergone hysterectomy were assigned into treatment group (*n* = 10) taking 50 mL of olive oil daily, or control group (*n* = 10) not receiving any form of supplementation. After one year of follow-up, bone mineral density of L3, L4 and left femur decreased significantly in the control group, but not in the treatment group [29]. The sample size of this study was small and subjected to bias because the control group did not receive placebo. 

Mazzanti et al. randomly divided 60 post-menopausal Caucasian women aged 50–61 years attending osteoporosis screening in a hospital into two groups. One group took 20 mL/day enriched extra virgin olive oil (vitamin K1 0.07 mg/100 mL, vitamin D3 50 µg/100 mL and vitamin B_6_ 6 mg/100 mL) and another group took 20 mL/day plain extra virgin olive oil. The enriched olive oil decreased the level of oxidative products and increased the antioxidant capacity of the plasma significantly compared to plain olive oil. Supplementation of enriched olive oil also decreased the level of undercarboxylated osteocalcin and ratio of undercarboxylated to carboxylated osteocalcin significantly compared to plain olive oil [38]. This was presumably contributed by vitamin K in the enriched oil, which is known to be involved in the carboxylation of glutamate residues in protein to form gamma-carboxyglutamate [39]. However, there was no control group taking placebo in this study, thus the effects of extra virgin olive oil per se could not be determined. The effects of olive oil on bone in human have been summarized in Table 2.

## 7. Future Research Directions

There is an abundance of animal studies showing that olives, olive oil and olive polyphenols are effective in preventing bone loss in post-menopausal osteoporosis models. A closer scrutiny of the results from some animal studies revealed that treatment of olive or its products improved bone mineral density but this did not translate into an increased biomechanical strength [26,31,32]. Reduced bone strength and the resultant fragility fractures are the ultimate consequences of osteoporosis. Changes in bone mineral density caused by treatment might precede changes in bone strength [15]. A larger dose or a longer treatment period might be required to have a significant impact on biomechanical strength in these animals.

In several studies it was observed that olive or its products were more effective in ovariectomized rats with inflammation than in those without inflammation [26,27,31,32]. This suggests that the bone protective actions of olive and its products are predominantly exerted through its anti-inflammatory actions rather than its oestrogenic actions at the doses used in these studies. As osteoblasts and macrophages are important sources of inflammatory cytokines in the bone, the anti-inflammatory mechanism of olive and its derivatives can be further elucidated in osteoblast and macrophage culture.

Oestrogen deficiency due to menopause is the most important cause of osteoporosis in women [7]. Phenolic compounds possessing oestrogenic effects might be potential osteoprotective agents in preventing post-menopausal bone loss. A study by Keiler et al. showed that phenolic content of olive oil was able to influence oestrogen-responsive genes in the uterus [34]. Future studies to identify the active components with the most prominent oestrogenic effects on bone and their interaction with oestrogen receptors on bone cells should be conducted. Oestrogen replacement therapy (oestrogen plus progestin) has been shown to reduce risk for hip fracture but increase risk for certain cancers and cardiovascular events in Women’s Health Initiative [40]. Thus, the safety of the oestrogenic activity of olive phenolic extract should be examined thoroughly because its binding to oestrogen receptors might not be organ-specific; thus long term consumption might aggravate disease sensitive to oestrogens. However, the oestrogen receptor binding affinity of phytoestrogens is generally lower compared to oestrogens [41]. 

The bone protective effects of olives, olive oil and its polyphenols have not been demonstrated in other bone loss models apart from oestrogen-deficiency model. Their effects on an animal model of osteoporosis due to testosterone deficiency should be conducted. Despite having a lower prevalence of osteoporosis, 40% of the osteoporotic hip fractures occur in men [2]. Male fracture patients also suffered from higher morbidity and mortality rate compared to female [42,43]. Thus, it is important to know whether men can benefit from dietary intervention with olive and its derivatives.

The rat model of osteoporosis is a valuable screening model of bone protective agent but it has several limitations. Rats do not undergo menopause so ovariectomy is necessary to induce post-menopausal bone loss similar to that seen in humans. Post-menopausal bone loss in humans also has an underlying chronic inflammatory factor, which was induced by administration of talc in some studies included in this review [19,26,27,31,32]. Rats reach skeletal maturity at approximately 12 months old. Thus, the use of a younger age rat model, as in some of the studies included in this review [26,27,29], does not mimic senile osteoporosis model. Instead, it resembles human having a lower peak bone mass (stunned skeletal growth) (reviewed in [44,45]). Further studies experimenting on rats should consider using skeletal mature old female rats subjected to ovariectomy that resembles post-menopausal osteoporosis in women.

There are some evidence of olive polyphenol extracts in promoting proliferation of pre-osteoblasts and mineralization of osteoblasts [33,35]. They also suppressed the formation of osteoclast-like cells in culture [33]. Oleuropein was noted to promote osteoblastogenesis but suppresses adipogenesis in bone marrow culture by modulating the expression of related downstream genes [37]. However, studies on the effects of other individual polyphenols on bone cells are limited. The upstream molecular pathways for the osteoblastogenesis-promoting and osteoclastogenesis-suppressing effects of olive polyphenols have not been illustrated clearly. It is not known whether bone protecting effects of olive polyphenols were the cumulative effect of antioxidant and anti-inflammatory pathways, or whether other pathways are involved. As suggested by Gracia-Martinez et al. [35], there might be other unknown compounds responsible for the bone protecting effects of olive oil extract because the total polyphenol content did not correlate with the efficacy in promoting pre-osteoblast proliferation.

Olive oil trials on bone health in humans could be improved by employing larger sample sizes and longer treatment periods. The two studies included in this review used bone turnover markers as the determinants of bone health and a small study used bone mineral density [21,29,38]. While it is acknowledged that changes in bone turnover markers could be observed in a shorter period of time, the improvement of bone health in patients ultimately lies on a positive change in bone mineral density. Proper randomization and blinding should be performed to avoid any bias in future trials on olive oil.

## 8. Conclusions

Olives, olive oil or olive polyphenols have the potential to be developed as bone protective agents. This is supported by evidence derived from preclinical studies using animal models of osteoporosis and a limited number of human studies. The bone protective effects of olive and its products are attributed to their ability to increase bone formation and inhibit bone resorption, by suppressing oxidative stress and inflammation. However, the exact pathways are still elusive and await future validation. Well-planned randomized controlled trials on olive and its derivatives are warranted to justify its use in osteoporosis prevention.

## Figures and Tables

**Table 1:** Animal studies on the bone protective effects of olives, olive oil and its polyphenols.

No.	Reference	Treatment; Dose; Length	Method of Inducing Bone Loss	Bone Mineral Density	Histology/ Histomorphometry	Bone Turnover Marker	Bone Strength	Oxidative Stress	Inflammation
1	Puel et al. 2007 [26]	Black Lucques olives; 6 g per day; 84 days	OVX or OVX + talc	↑ (diaphyseal)	NA	↔	↔	↔	↔
2	Puel et al. 2004 [27]	Extra virgin olive oil; 50 g/kg diet; 80 days. Oleuropein; 0.15 g/kg diet; 80 days	OVX or OVX + talc	↑	NA	↔	↑	↔	↓ (olive oil only)
3	Saleh and Saleh 2011 [28]	Olive oil; 1 mL/100 g diet; 12 weeks	OVX	↑ bone thickness	NA	↓ calcim and ALP activity	NA	↓	NA
4	Liu et al. 2014 [29]	Olive oil; 1 mL/100 g diet; 12 weeks	OVX	↑	NA	↔	NA	↓	NA
5	Puel et al. 2006 [32]	Oleuropein; 2.5–15 mg/kg; 100 days	OVX or OVX + talc	↑	NA	↓ formation & resorption markers	↔	↔	NA
6	Puel et al. 2008 [31]	Tyrosol 0.017%, or hydroxytyrosol 0.017%, or olive mill wastewater 0.17%, or olive mill wastewater extract 0.08% or 0.0425%; 84 days.	OVX or OVX + talc	↑ (all except OMWW)	NA	↑ osteocalcin; ↔ DPD	↔	↓ isoprostane; ↔ FRAP	↓ granulocytes (OMWW 0.0425%); ↔ fibrinogen
7	Hagiwara et al. 2011 [33]	Hydroxytyrosol or tyrosol or oleuropein; 10 mg/kg; 28 days	OVX	↑ trabecular; ↔ cortical	NA	NA	NA	NA	NA
8	Keiler et al. 2014 [34]	Olive oil phenolic extract; 800 mg/kg diet; 12 weeks	OVX	NA	↔	NA	NA	NA	NA

Abbreviation: ALP = alkaline phosphatase; DPD = deoxypyridinoline; FRAP = ferric reducing ability of plasma; NA = data not available; OMWW = olive mill waste water; OVX = ovariectomy; talc = talc-induced inflammation; Legend: ↑ indicates a significant increase, ↓ a significant decrease and ↔ an insignificant change compared to control animals.

**Table 2:** Human studies on the bone protective effects of olive oil.

No.	Reference	Subjects and Treatment	Bone Mineral Density	Bone Turnover Markers	Antioxidants
1	Fernandez-Real et al. 2012 [21]	127 community-dwelling men aged 55–80 years participated in the Prevencion con Dieta Mediterranea (PREDIMED) study with high cardivascular risk. They were divided into 3 groups: Mediterranean diet with virgin olive oil, Mediterranean diet with mixed nuts, low-fat diet. 2 years follow up.	NA	↑	NA
2	Liu et al. 2014 [29]	Patients aged 30–50 years who had undergone a hysterectomy. 10 took 50 mL olive oil daily and 10 took nothing. Follow up 1 year.	↑	↑	NA
3	Mazzanti et al. 2015 [15]	60 Caucasian post-menopausal women aged 50–61 years attending health screening for osteoporosis in a hospital. They were randomly divided into two groups, 1 taking enriched extra virgin olive oil (vitamin K1 0.07 mg/100 mL, vitamin D3 50 µg/100 mL and vitamin B_6_ 6.0 mg/100 mL) and another taking plain virgin olive oil for 1 year.	NA	↑	↑

Legend: ↑ indicates a significant improvement; NA = data not available.
